# Ultra-Fast Displaying Spectral Domain Optical Doppler Tomography System Using a Graphics Processing Unit

**DOI:** 10.3390/s120606920

**Published:** 2012-05-25

**Authors:** Hyosang Jeong, Nam Hyun Cho, Unsang Jung, Changho Lee, Jeong-Yeon Kim, Jeehyun Kim

**Affiliations:** 1 School of Electrical Engineering and Computer Science, Kyungpook National University, 1370, Sankyuk-dong, Buk-gu, Daegu 702-701, Korea; E-Mails: hyosangj@gmail.com (H.J.); nhcho@knu.ac.kr (N.H.C.); cester@paran.com (U.J.); song31037@knu.ac.kr (C.L.); 2 Division of General Studies, Ulsan National Institute of Science and Technology, Ulsan 689-798, Korea; E-Mail: jkim@unist.ac.kr

**Keywords:** ODT, OCT, GPU, real-time, CUDA

## Abstract

We demonstrate an ultrafast displaying Spectral Domain Optical Doppler Tomography system using Graphics Processing Unit (GPU) computing. The calculation of FFT and the Doppler frequency shift is accelerated by the GPU. Our system can display processed OCT and ODT images simultaneously in real time at 120 fps for 1,024 pixels × 512 lateral A-scans. The computing time for the Doppler information was dependent on the size of the moving average window, but with a window size of 32 pixels the ODT computation time is only 8.3 ms, which is comparable to the data acquisition time. Also the phase noise decreases significantly with the window size. Since the performance of a real-time display for OCT/ODT is very important for clinical applications that need immediate diagnosis for screening or biopsy. Intraoperative surgery can take much benefit from the real-time display flow rate information from the technology. Moreover, the GPU is an attractive tool for clinical and commercial systems for functional OCT features as well.

## 1. Introduction

Optical Coherence Tomography (OCT) has been widely accepted as a non-invasive high resolution imaging modality for *in vivo* biological specimens [1]. After the emergence of spectral domain OCT (SD-OCT) and swept source OCT (SSOCT), the real time imaging and displaying feature has become one of the major competing categories [2–4]. Real time displaying feature is another key parameter to deliver the OCT technology to clinical and industrial fields because dynamic changes of the targets are often screened by human vision. Current real-time video-rate display is commonly limited to displaying OCT intensity images. Phase information related to flowing objects requires a significant post-processing effort, and also most flow dynamics require high speed acquisition to trigger certain interesting events. The need for real-time display of Doppler frequency shift images is urgently required for monitoring flow samples, but such a feature has not been reported yet.

Recent commercialization for massive parallel processing units provides an easily adaptable solution to this problem. Recently developed Graphics Processing Units (GPUs) enable very fast processing of OCT signals, but also can execute parallel, general purpose numerical solutions surpassing the use of CPUs. Several years ago NVIDIA Corporation presented a parallel computing architecture named Compute Unified Device Architecture (CUDA) commonly adapted in computer games for more natural presentation of sceneries. Use of GPUs in the OCT research field for fast display of intensity images has been reported several times [5–8]. In this paper, we report a novel ultra-fast displaying Spectral Domain Optical Doppler Tomography (SD-ODT) by use of CUDA processes for real-time display of OCT intensity and Doppler images simultaneously at a frame rate of 120 fps for a 2,048 × 512 pixel image size.

## 2. System Configuration and Signal Processing Architecture

**Figure 1.**
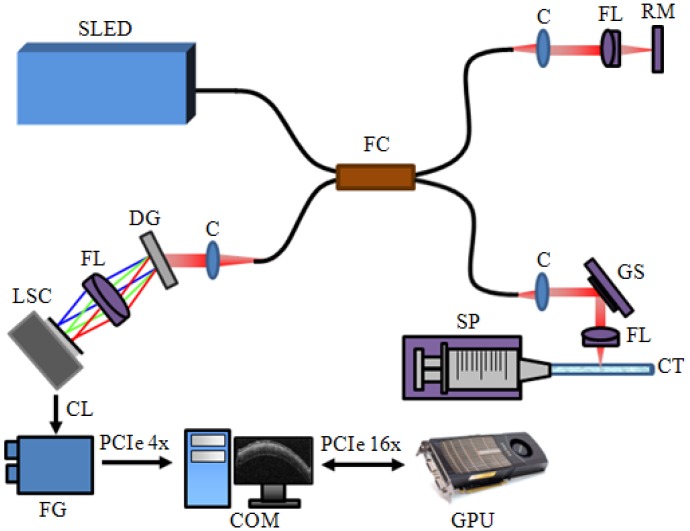
Schematic of the SD-ODT system. C: Collimator. FL: Focusing lens. RM: Reference mirror. FC: 2 × 2 Fiber coupler. LSC: CMOS Line scan camera. DG: Diffraction grating. GS: Galvanometer scanner. SP: Syringe pump. CT: 750 μm Capillary tube. PCIe: PCI express. FG: Frame grabber. COM: Host computer.

The schematic diagram of the developed SD-OCT system is shown at Figure 1. A 12-bit CMOS line scanning camera (Sprint spL2048-140 k, Basler AG) with 70,000 line/s effective line rate at 2,048 pixel mode was used as the detector of the SD-OCT system. The transmission type diffraction grating (Spatial Frequency 1,800 lpmm, Nominal AOI/AOD 46.05 Degrees, Wasatch Photonics) was adapted to enhance light efficiency in the detection path. Combined with a superluminescence diode (SLED) (λ_0_ = 850 nm, Δλ = 55 nm, Exalos AG) as a light source, a fiber-based interferometer was implemented. The light source was split into sample and reference arms with the latter terminated by a stationary mirror. A probe at the end of the sample arm delivered light to a sample and collected back-scattered light from different depths in the sample. B-mode scanning was performed using a galvanometer scanning mirror (GVS002, Thorlabs) at the back focal plane of the objective lens at the sample arm. The developed system with the axial and lateral resolutions of 4 μm and about 12 μm, respectively. The measured depth range was 4 mm.

A scattering particle filled capillary tube with a 750 μm diameter was used to demonstrate the flow rate imaging feature. A syringe pump (Harvard Apparatus, accuracy ±0.5%, flow rate maximum 7.909 mL/mm, flow rate minimum 0.0014 μL/h) provided changes of the flow rate in the tube. The detected OCT signals were transferred to a host memory in the PC (Personal Computer) mounted with six CPUs (Core i7 980X Hexa cores, 3.33 GHz Clock rate, Intel) through a frame grabber (PCIe-1433, 850 MB/s Bandwidth, National Instruments, USA). The galvanometer scanning mirror was driven by the PC with a data acquisition board (PCIe-6321, National Instruments). The PC also contained a graphics card (Geforce GTX480, 700 MHz Clock rate, 480 CUDA processor, NVIDIA).

In order to apply the GPU technology to both OCT and ODT signal processing, an optimum combination of CPU and GPU is designed. Two separate buffers are allocated in the host memory of CPU and they are assigned as CPU thread one. These buffers are mainly dedicated for temporal data storage right after data acquisition. Due to the limited data transfer channel between CPU and GPU careful data handling was necessary to minimize any bottleneck events during the data transfer. We report a significant decrease in the processing time when we host memory to allocate the acquired data. The conventional method utilizing the memory in the frame grabber measures 16 ms processing for 2,048 × 512 pixel data size compared to 8.3 ms of the proposed two-separate buffer method. As a device memory in the GPU, the signal processing job is divided into 480 CUDA processors.

**Figure 2.**
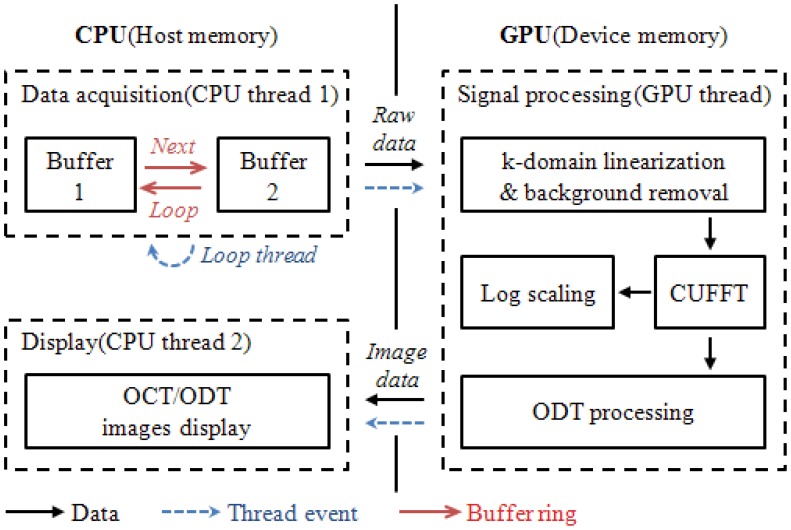
Architecture of the SD-ODT system with signal processing part implemented in a GPU.

Figure 2 displays the data flow chart for the system including the flow of data path, thread events, and the buffer ring. First the data acquisition thread stored incoming two dimensional signals into the first buffer allocated in the host memory and called a signal processing thread. Later, the self-iterated acquisition thread continuously transferred the incoming signals to the second buffer without any temporal delay between the acquisition events. The signal processing thread copied the frame data stored in the buffers of the host memory through the PCI express x16 2.0 interface into the device memory. Later, the processing divided 480 CUDA sub-processors to process further signal processing for OCT and ODT. K-domain linearization was completed using the full-range k-domain linearization [9]. The reconstructed OCT and ODT image were transferred back to the host memory to be displayed.

## 3. Doppler Frequency Shift

**Figure 3.**
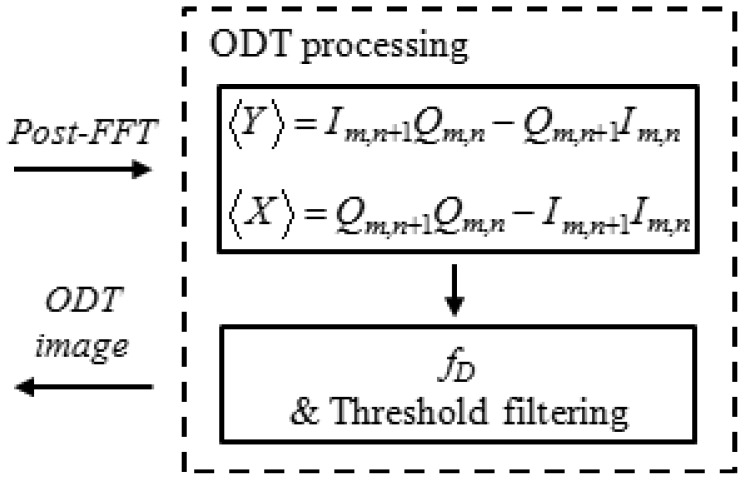
Flowchart of Doppler frequency shift calculation by a GPU.

The interference fringe pattern can include structural and phase information. After Fourier transformation of the complex signal *I* + *jQ*, the structural OCT image was calculated from Equation (1) [10,11]. The phase information was extracted from real and imaginary values and the Doppler frequency shift was calculated from Equation (2) [10–12]:

(1) $$S_{\text{OCT}}=\sqrt{I^{2}+Q^{2}}$$

(2) $$f_{D}=\frac{f_{a}}{2\pi }\tan^{-1}\left\{\frac{\frac{1}{\left(M\right)\left(N-1)\right)}\overset{M}{\underset{m=0}{\text{∑}}}\overset{N-1}{\underset{n=1}{\text{∑}}}\left[I_{m,n+1}Q_{m,n}-Q_{m,n+1}I_{m,n}\right]}{\frac{1}{\left(M\right)\left(N-1)\right)}\overset{M}{\underset{m=0}{\text{∑}}}\overset{N-1}{\underset{n=1}{\text{∑}}}\left[Q_{m,n+1}Q_{m,n}-I_{m,n+1}I_{m,n}\right]}\right\}$$

where *f_a_* is the sampling rate, and *I* and *Q* represent in-phase and quadrature phase information in the interference signal. *M* and *N* are the moving average window size for axial and lateral directions, respectively, whereas m and n is the data position in the window. Calculation of the kasai autocorrelation algorithm [13,14] was divided to two different steps for GPU processing as shown in Figure 3. Firstly, <X> and <Y> are calculated by Equation (3). All the OCT data is processed by parallel processing, because GPU computing is specialized for highly parallel computation. The moving average calculation can be processed after the Equation (3) is complete:

(3) $$\begin{array}{l} \langle Y\rangle =I_{m,n+1}Q_{m,n}-Q_{m,n+1}I_{m,n}\\ \langle X\rangle =Q_{m,n+1}Q_{m,n}-I_{m,n+1}I_{m,n} \end{array}$$

Then *M* × *N* window averaging and *f_D_* was evaluated using Equation (4) after cropping phase noise below a predefined threshold:

(4) $$f_{D}=\frac{f_{a}}{2\pi }\tan^{-1}\left\{\frac{\frac{1}{\left(M\right)\left(N-1)\right)}\overset{M}{\underset{m=0}{\text{∑}}}\overset{N-1}{\underset{n=1}{\text{∑}}}\langle Y\rangle }{\frac{1}{\left(M\right)\left(N-1)\right)}\overset{M}{\underset{m=0}{\text{∑}}}\overset{N-1}{\underset{n=1}{\text{∑}}}\langle X\rangle }\right\}$$

## 4. ODT Data Processing

**Figure 4.**
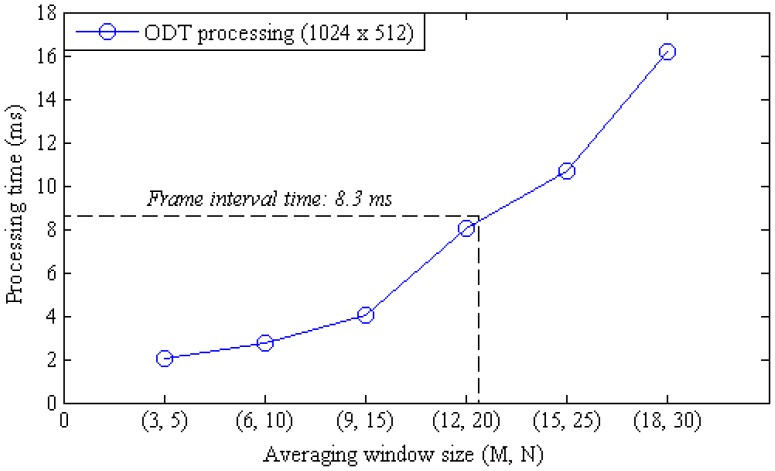
Comparison in ODT processing time between averaging window sizes.

**Figure 5.**
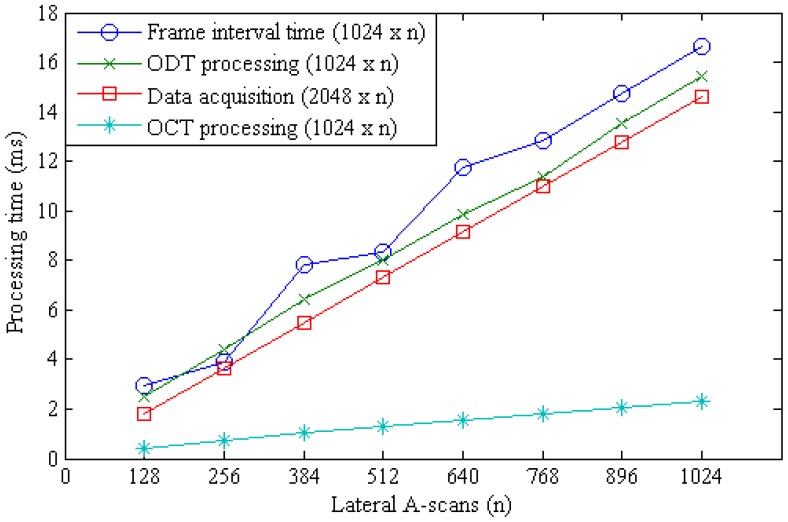
Comparison in ODT processing time between lateral A-scans numbers. Averaging window size: 12 + 20 (*M* + *N*).

The moving average calculation is still the most computation intensive procedure in the CUDA processing and the calculation time is linearly proportional to the window size. The effect of the moving average window size to the frame rate was examined by measuring the OCT and ODT processing time at different window sizes as shown in Figure 4. The time interval between frames for a 2,048 × 512 pixel sized data took 8.3 ms in the developed system. The frame interval time is determined by other processing threads than the moving average calculation when the window size is below 32 pixels. Figure 5 shows the processing time variation according to the change of the lateral scan size when the window size was 32 pixels.

**Figure 6.**
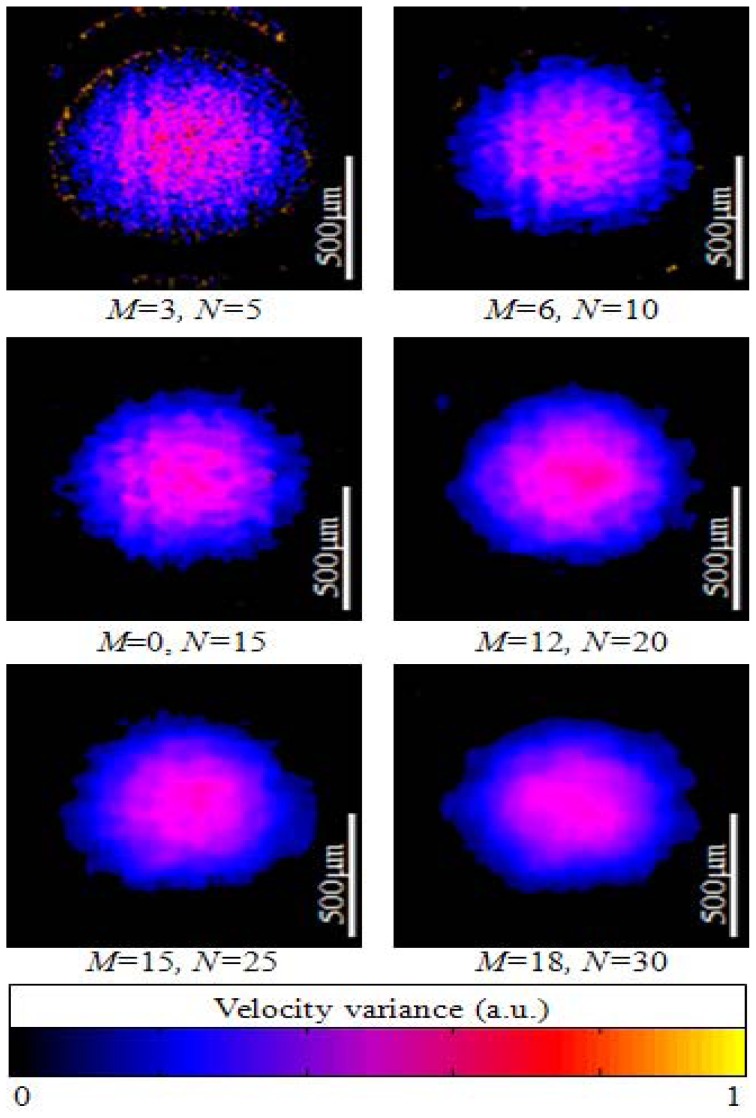
B-scan ODT images of flowing particles in a capillary tube with different averaging window sizes. Doppler angle: 80°. Flow velocity: 80 mm/s.

The resultant ODT images are shown at Figure 6 after B-mode scanning of the flowing particle filled tube at different averaging window sizes. ODT calculation results using CUDA also confirm that the window size is inversely proportional the phase noise as previously reported in the conventional ODT technique. The velocity variance each was calculated by using the four-quadrant arctan method and displayed color coded [12].

## 5. Results and ODT Imaging

**Figure 7.**
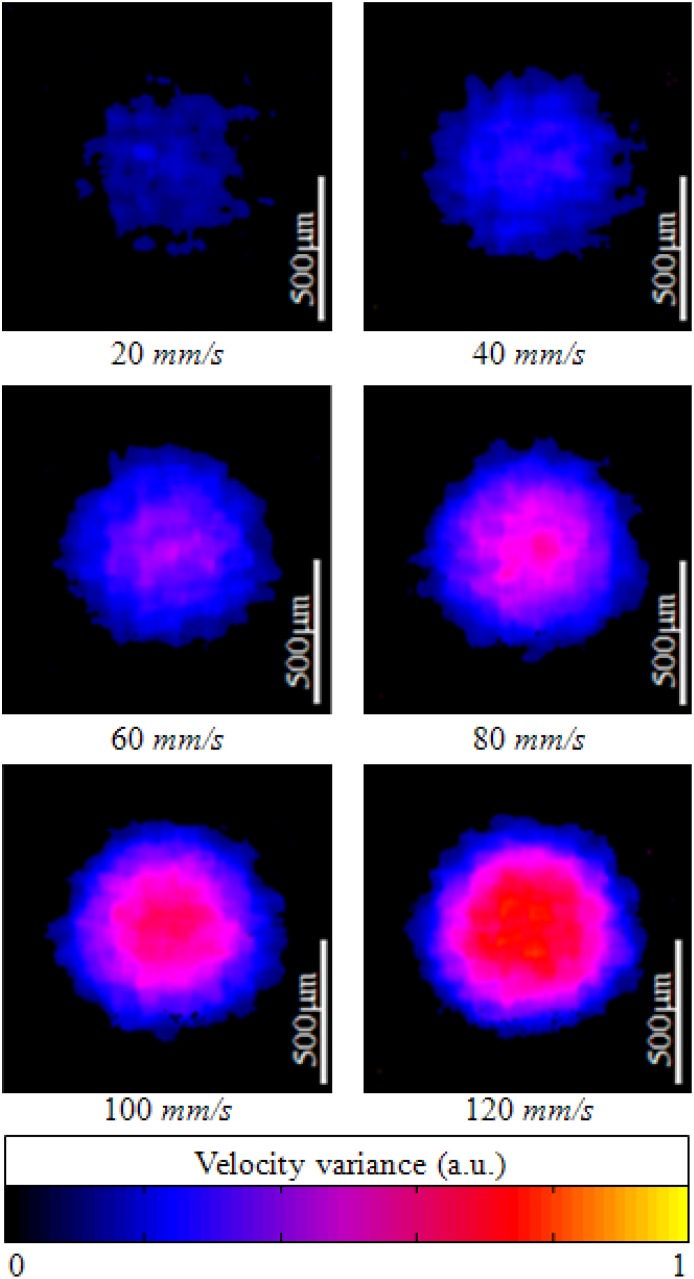
B-scan ODT images of capillary tube with different flow velocities. Doppler angle: 80°. Averaging window size: 12 + 20 (*M* + *N*).

**Figure 8.**
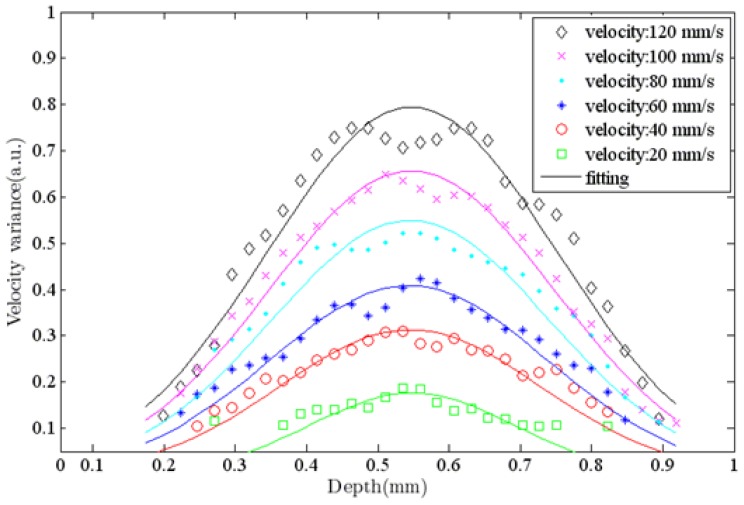
The velocity profile in the depth direction in the center of ODT images.

Figure 7 shows ODT images of a capillary tube with flowing particles at different flow rates. The average window size was 12 × 20 pixels in the axial and lateral direction, respectively. The ODT displaying frame rate was 120 fps for the image size of 1,024 × 512 pixels. The central velocity variance profiles at each flow rate (Figure 7) are calculated at Figure 8.

**Figure 9.**
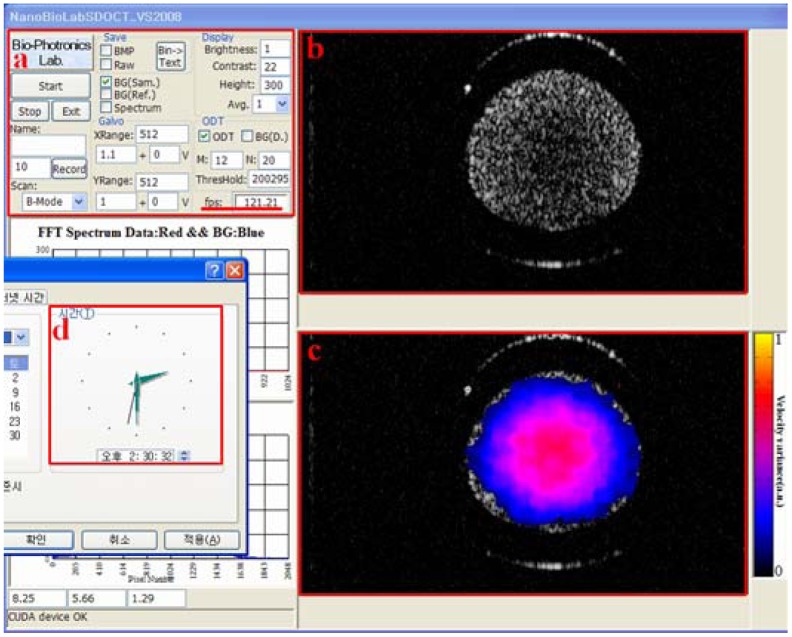
Captured movie of experiment for a capillary tube ODT. Doppler angle: 80°. Averaging window size: 12 + 20 (*M* + *N*). Displaying image size: 512 W × 300 H pixel. (**a**) System setup. Red line: actual frame rate; (**b**) OCT image; (**c**) ODT image; (**d**) The watch for time comparison.

Figure 9 shows the captured movie of experiment for OCT and ODT images of flowing particles in a capillary tube. The movie contains a scattering flow channel with different flow velocities from 0 to 120 mm/s with increase of 20 mm/s. The frame rate was calculated by averaging the total processing time for 500 frame displays including acquisition and display time. The average frame rate was 120 fps for simultaneous display OCT and ODT images.

## 6. Discussion and Conclusions

We demonstrate an ultrafast displaying SD-ODT using GPU computing. The calculation of FFT and Doppler frequency shift is accelerated by the GPU. The computing time for the Doppler information was dependent on the size of the moving average window, but for a window size of 32 pixels the ODT computing time is 8.3 ms, which is comparable with the data acquisition thread time. The phase noise also decreases significantly with size. Our system can display processed OCT and ODT images simultaneously in real time at 120 fps for 1,024 pixels × 512 lateral A-scans. Since the performance of a real-time display for OCT/ODT is very important for clinical applications that need immediate diagnosis for screening or biopsy, intraoperative surgery can benefit greatly from the real-time display flow rate information possible with this technology. Moreover, the GPU is an attractive tool for clinical and commercial systems for its functional OCT features as well.
